# Synthesis and Characterization of Graphene–Silver Nanoparticle Hybrid Materials

**DOI:** 10.3390/ma13204660

**Published:** 2020-10-19

**Authors:** Zoltán Osváth, András Pálinkás, Gábor Piszter, György Molnár

**Affiliations:** Centre for Energy Research, Institute of Technical Physics and Materials Science, P.O. Box 49, 1525 Budapest, Hungary; andras.palinkas@energia.mta.hu (A.P.); gabor.piszter@energia.mta.hu (G.P.); gyorgy.molnar@energia.mta.hu (G.M.)

**Keywords:** silver nanoparticles, local surface plasmon resonance, graphene, hybrid nanostructures, scanning tunneling microscopy, tunneling spectroscopy, atomic force microscopy, UV-Vis spectroscopy

## Abstract

Silver nanoparticles (Ag NPs) play important roles in the development of plasmonic applications. Combining these nanoparticles with graphene can yield hybrid materials with enhanced light–matter interaction. Here, we report a simple method for the synthesis of graphene–silver nanoparticle hybrids on highly oriented pyrolytic graphite (HOPG) substrates. We demonstrate by scanning tunneling microscopy and local tunneling spectroscopy measurements the electrostatic *n*-type doping of graphene by contact with silver. We show by UV-Vis reflectance investigations that the local surface plasmon resonance (LSPR) of Ag NPs partially covered with graphene is preserved for at least three months, i.e., three times longer than the LSPR of bare Ag NPs. The gradual loss of LSPR is due to the spontaneous sulfurization of non-covered Ag NPs, as revealed by scanning electron microscopy and energy-dispersive X-ray spectroscopy. We show that the Ag NPs completely sandwiched between graphene and HOPG do not sulfurize, even after one year.

## 1. Introduction

Noble metallic nanoparticles (NPs) are extensively applied for chemical and biological sensing due to their local surface plasmon resonance (LSPR) [1,2] and surface-enhanced Raman scattering (SERS) properties [3,4]. In particular, the optical properties of gold (Au) and silver (Ag) nanoparticles are highly investigated due to their enhanced interaction with light [5,6,7,8,9,10]. Their LSPR can be tuned by controlling the size, shape, dispersion, and uniformity of the NPs, and also the dielectric constant of the surrounding medium [11,12,13,14,15,16,17]. Nanostructured Ag is the best material for plasmonics due to the absence of interband absorptions and low optical loss at optical frequencies [18]. However, silver has poor stability under ambient conditions, forming Ag_2_S on its surface. This leads to morphological changes of the NPs and significant diminishing of the optical properties [19]. Preserving the high surface plasmon resonance intensity of silver nanoparticles is of key importance in potential applications. The most common approach to improve the chemical stability of Ag nanostructures is to form core–shell structures by passivating the surface of Ag with a protective shell, which can be either organic or inorganic (see [20] for a recent review). This coating should be at the same time thick enough to fully protect Ag NPs and thin enough to conserve the strong near-field interaction in SERS-based sensing experiments. In this respect, graphene seems to be the ideal protective coating [21], since it is atomically thin and impenetrable to standard gases, including helium [22,23]. Nevertheless, for large-area graphene grown by chemical vapor deposition (CVD), the oxygen can infiltrate through defects and grain boundaries [24].

The synthesis of Ag NPs can be realized over a wide range of strategies [25], depending on the required shape of the nanoparticles. The most commonly used method is the chemical reduction in a bottom-up approach. The size and shape of produced Ag NPs depend on many factors, such as the temperature, the concentration of the silver precursor, and the strength of chemical interaction between the capping agent and various crystallographic planes of silver [26]. In this work, we present a simple method for the preparation of Ag NPs and graphene–silver nanoparticle hybrids directly onto highly oriented pyrolytic graphite (HOPG) substrates. The morphology, optical, and electronic properties of the hybrid nanomaterials are investigated by atomic force microscopy (AFM), UV-Vis reflectance spectroscopy, and scanning tunneling microscopy (STM) and scanning tunneling spectroscopy (STS), respectively. Electron transfer from silver to graphene is observed by local STS measurements on graphene-covered silver nanostructures. We show that a graphene overlayer can preserve the LSPR of Ag NPs, however its protective efficiency is limited by the area of graphene coverage. We demonstrate by scanning electron microscopy (SEM) and energy-dispersive X-ray spectroscopy (EDX) that the Ag NPs completely sandwiched between graphene and HOPG are protected from sulfur, even 14 months after preparation.

## 2. Materials and Methods

Bulk silver of 99.99% purity (Metal-Art Zrt., Budapest, Hungary) was placed in an electrically heated tungsten boat for evaporation. Thin silver films of 7 nm nominal thickness were evaporated onto HOPG substrates at a background pressure of 5 × 10^−7^ mbar and rate of 0.1 nm s^−1^. During the process, the substrate remained at room temperature. Immediately after silver deposition and opening of the vacuum chamber, the thin silver films were covered with CVD graphene, which was synthesized as described in a previous paper [27]. For the transfer of large-area graphene samples grown on copper foil, we applied a polymer tape. The copper foil was etched using a mixture of CuCl_2_ aqueous solution (20%) and HCl (37%) in a 4:1 volume ratio. The tape holding the graphene was rinsed in distilled water, dried, and pressed onto the deposited silver thin film. Graphene-covered silver thin films were obtained by lifting the tape with tweezers. In order to drive the surface diffusion of deposited silver and to form the Ag nanoparticles, subsequent annealing of both bare and graphene-covered thin silver films was performed at 400 °C under inert gas (Ar) atmosphere for 90 min. We used these annealing parameters, which worked earlier for the preparation of gold nanoparticles, as reported recently [27].

The Ag NPs and graphene–Ag NP hybrid structures were investigated by tapping mode AFM measurements performed on a MultiMode 8 (Bruker France S.A.S, Champs sur Marne, France), along with STM and STS measurements using a DI Nanoscope E (Bresso, Italy) operating under ambient conditions. The optical reflectance properties of the samples were measured in the wavelength range of 200 to 1000 nm using an Avantes AvaSpec-HS1024 × 122TEC fibre optic spectrometer (Avantes BV, Apeldoorn, The Netherlands). We used a bifurcated probe for illumination and detection with 200 μm core diameters. The reflectance spectra of the samples were recorded by collecting the specular reflected light under normal incidence illumination with an Avantes AvaLight DH-S-BAL balanced UV-Vis light source (Apeldoorn, The Netherlands). Scanning electron microscopy (SEM) and energy-dispersive X-ray spectroscopy (EDX) investigations were performed with Thermo Scientific Scios2 (Brno, Czech Republic) and Oxford X-max 20 (Oxford, UK) instruments, respectively.

## 3. Results and Discussion

**Figure 1:**
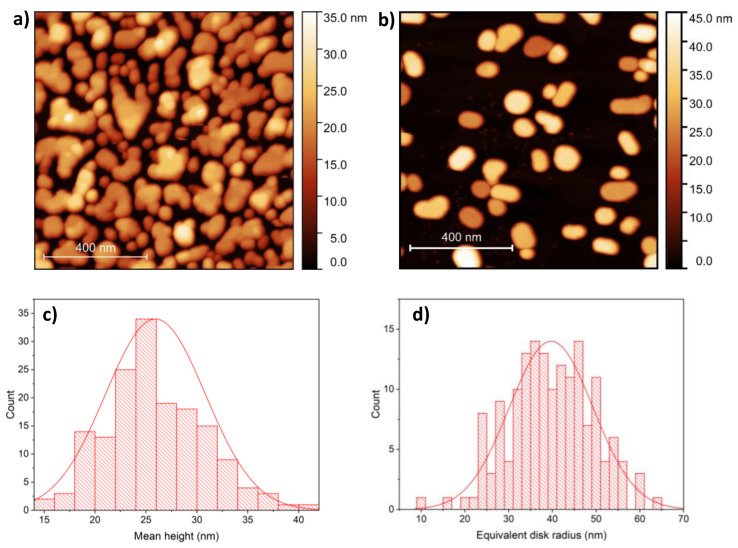
(**a**) Atomic force microscopy (AFM) image of 7 nm Ag as-deposited onto highly oriented pyrolytic graphite (HOPG) substrate. (**b**) AFM image of the same sample as in (**a**) after annealing at 400 °C. Mean height (**c**) and equivalent disc radius (**d**) distribution of 161 Ag NPs formed during annealing.

The surface of 7 nm Ag deposited onto HOPG is shown in Figure 1a, as measured by AFM. The as-deposited thin film is not continuous and can be characterized by a root mean square roughness (RMS) of 7.5 nm. Annealing at 400 °C resulted in flat nanoparticles (Figure 1b) formed due to the surface diffusion and aggregation of silver clusters. The nanoparticles can be characterized by having a mean height of 26 ± 4.9 nm (Figure 1c) and equivalent disk radius of 40 ± 9.6 nm (Figure 1d). We applied this method earlier for the preparation of flat gold nanoparticles with similar dimensions and surface coverage [26].

**Figure 2:**
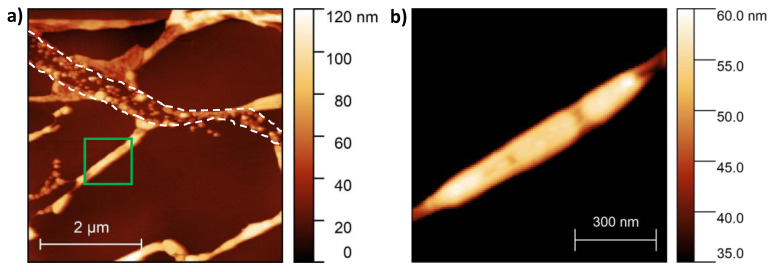
(**a**) AFM image of graphene-covered Ag nanostructures formed on HOPG substrate during annealing at 400 °C. Bare nanoparticles are also observed between the two white dashed lines, which mark a discontinuity of the graphene overlayer. (**b**) Larger magnification of AFM image corresponding to the square marked with green lines in (**a**), showing graphene–Ag NP–HOPG sandwich structure.

We also prepared graphene-covered samples by transferring the two-dimensional carbon sheet onto the as-deposited silver thin film. Typical AFM images of the graphene–Ag hybrid nanostructures obtained after a similar annealing procedure are shown in Figure 2. Elongated structures, as well as large, nanoparticle-free graphene–HOPG areas, are also observed (Figure 2a). Closer investigation of the elongated structures reveals graphene-covered groups of Ag NPs, as shown in Figure 2b (graphene–Ag NP–HOPG sandwich structure).

**Figure 3:**
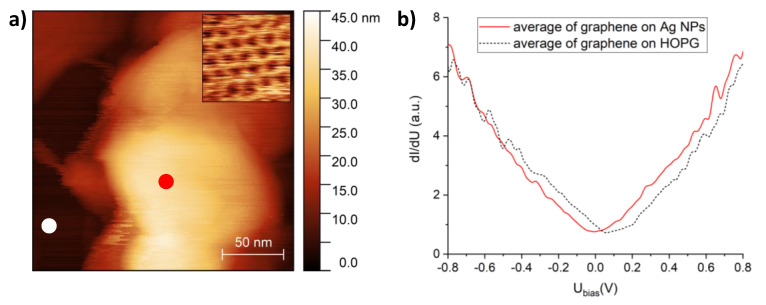
(**a**) Scanning tunneling microscopy (STM) image of a graphene-covered Ag nanostructure. Dark-colored regions correspond to graphene on the HOPG substrate. Tunneling parameters: I = 0.4 nA, U = 0.8 V. Atomic-resolution STM image of silver-supported graphene is shown in the inset. (**b**) The dI/dU spectra measured on graphene–HOPG (dashed line) and graphene–Ag (red line). The STS measurements were performed at the graphene–HOPG and graphene–Ag positions marked in (**a**) with white and red symbols, respectively.

Graphene–Ag NPs were also investigated by STM and STS in order to study the effect of silver contact on the density of electronic states of graphene. Figure 3a shows a STM image of a graphene-covered Ag nanostructure with a maximum height of 45 nm. The atomic-resolution inset image reveals the honeycomb lattice of the graphene overlayer. STS measurements were performed both on the top of the nanostructure (red symbol in Figure 3a) and on nearby graphene–HOPG (Figure 3a, white symbol). The dI/dU spectra shown in Figure 3b were obtained by numerically differentiating and averaging 25 different current (I)–voltage (U) characteristics for each area. A clear shift is observed between the two average dI/dU spectra, with *p*-doped graphene on HOPG (Dirac point around 70 mV) and slightly *n*-doped graphene on the Ag NP (Dirac point around –10 mV). This local probe measurement is in agreement with previous results, where electron transfer from Ag NPs to graphene was demonstrated by Raman spectroscopy [28].

**Figure 4:**
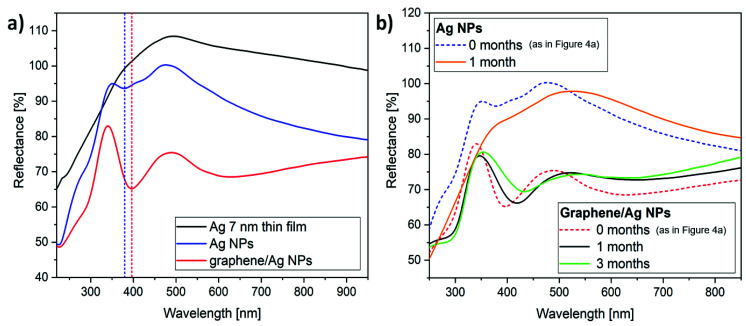
(**a**) Optical reflectance spectra of the as-deposited Ag thin film (black), the Ag NPs produced by annealing (blue), and the graphene-covered Ag NPs (red). The LSPR of Ag NPs (blue dashed line) is redshifted when covered with graphene (red dashed line). (**b**) Optical reflectance spectra measured after one month on bare Ag NPs (orange) and on graphene–Ag NPs (black). For better comparison, the initial spectra from (**a**) are also shown (blue dashed and red dashed lines, respectively). The spectrum of graphene–Ag NPs measured after 3 months is also plotted (green).

**Figure 5:**
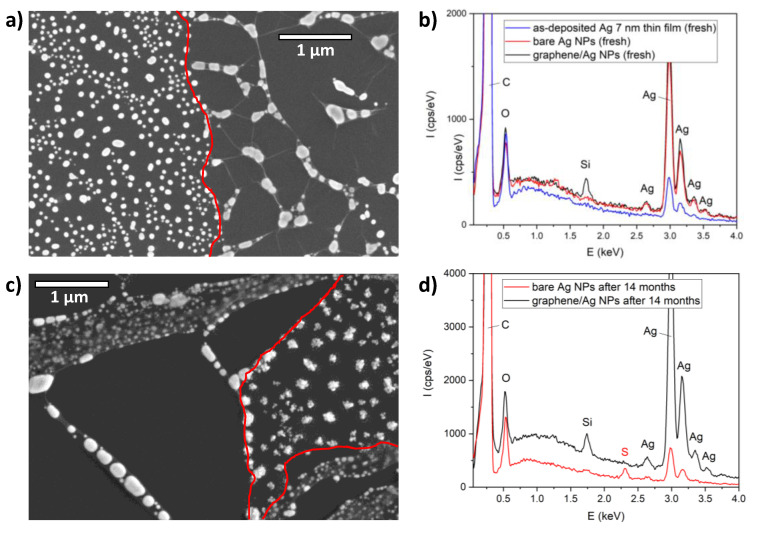
(**a**) SEM image of freshly prepared Ag NPs partially covered with graphene. The graphene edges are marked with red lines as guides for the eye. (**b**) EDX spectra measured on freshly prepared 7 nm Ag thin film (blue), bare Ag NPs (red), and graphene–Ag NPs (black). (**c**) SEM image of Ag NPs partially covered with graphene, 14 months after preparation. Graphene edges are marked with red lines as guides for the eye. (**d**) EDX spectra measured on bare Ag NPs (red) and graphene–Ag NPs (black), 14 months after preparation.

Next, we discuss the optical properties of the prepared samples. The reflectance spectra of the as-deposited Ag thin film (Figure 1a), bare Ag NPs (Figure 1b), and graphene-covered Ag NPs (Figure 2) are shown in Figure 4a. While the spectrum of the as-deposited Ag thin film is featureless, we observe a reflectance minimum at 379 nm for the sample with bare Ag NPs, which is attributed to the LSPR of the nanoparticles. The LSPR is more pronounced for the graphene-covered Ag NPs, which is redshifted to 396 nm. This redshift can be partly induced by the increased effective refractive index of the medium, due to the presence of a graphene overlayer [29]. On the other hand, the LSPR frequency ($\omega_{LSPR}$) is closely related to the bulk plasmon frequency ($\omega_{P}$) of the metal through [30]:

(1) $$\omega_{LSPR}\approx \frac{\omega_{P}}{\sqrt{1+2\varepsilon_{m}}}=\sqrt{\frac{Ne^{2}/m\varepsilon_{0}}{1+2\varepsilon_{m}}}$$

where N is the density of electrons in the NPs, e is the electronic charge, m is the effective mass of the electron, and $\varepsilon_{0},\;\varepsilon_{m}$ are the permittivity of free space and the surrounding medium, respectively. The transfer of electrons from Ag NPs to graphene, as demonstrated in Figure 3, decreases N and also induces a redshift of the LSPR wavelength. In addition, possible electrostatic coupling [31] between closely spaced graphene-encapsulated Ag NPs, as well as the formation of larger NPs (see Figure 5a) [32], can also contribute to the observed total LSPR redshift of 17 nm. The reflectance decreased at all wavelengths compared to the reflectance from bare Ag NPs, which is attributed to the enhanced light absorption of graphene [33].

Further, we investigated how the optical properties of Ag NPs and graphene–Ag NPs kept under ambient conditions vary in time. We performed the same reflectance measurements on the same samples one month and three months after preparation. The corresponding spectra are shown in Figure 4b. It can be clearly observed that due to spontaneous sulfurization, the optical reflectance spectrum of bare Ag NPs already loses its features after one month, similar to earlier reports [19,21]. In contrast, graphene-covered Ag NPs have well-defined LSPR, even after three months. This is in agreement with the Raman spectroscopy data reported very recently [34], which show the stability of graphene-covered Ag NPs after 10 weeks. Nevertheless, the amplitude of the resonance decreases and the LSPR gradually shifts towards larger wavelengths, i.e., the reflectance minimum is observed at 418 and 433 nm after one and three months, respectively. Further reflectance measurements revealed that the LSPR of graphene-covered Ag NPs vanished in approximately nine months after preparation.

For a more detailed study of spontaneous sulfurization, we investigated the graphene-covered Ag NPs by SEM and EDX. The SEM image of freshly prepared and partially covered Ag NPs is shown in Figure 5a. The edge of the graphene is marked with red lines as guides for the eye. The left part of the image shows bare Ag NPs similar to the ones measured by AFM (Figure 1b), while we can observe several graphene-encapsulated groups of Ag NPs (as in Figure 2b) on the right part of the image. Nanoparticles with similar shape and size can be observed in both non-covered and graphene-covered areas. However, due to the confinement induced by the graphene overlayer, the encapsulated NPs are closer to each other. Moreover, they tend to coalesce and to form larger nanoparticles. EDX analysis of bare and graphene-encapsulated NPs show characteristic Ag peaks near 3 keV, as shown in Figure 5b. Additionally, on graphene-covered areas, a Si peak is observed at 1.74 keV (Figure 5b, black), which is probably due to contamination during graphene transfer (it is missing on areas with bare Ag NPs). For comparison, the EDX spectrum of the freshly evaporated 7 nm Ag thin film is also displayed. The same measurements were performed on samples kept under ambient conditions for 14 months, as shown in Figure 5c,d. Figure 5c shows nanoparticles partially covered with graphene. Non-covered nanoparticles are observed in the right part of the image, between the two red lines marking the graphene edges. It is clear that the structure of nanoparticles changed remarkably and they have a less-defined shape after 14 months. In contrast, the graphene-encapsulated NPs observed on the lower left part of Figure 5c have the same shape as freshly prepared NPs. EDX analysis of aged non-covered nanoparticles (Figure 5d, red) reveals the presence of sulfur (peak at 2.3 eV), which is the spectroscopic signature of spontaneous sulfurization from air. 

Importantly, no sulfur is observed on graphene-encapsulated Ag NPs (Figure 5d, black), even after 14 months. Here, we have to stress that the total graphene coverage of graphene-coated samples is 40–50% as a result of the transfer process (graphene breaks easily at grain boundaries). This infers that the gradual loss of plasmonic properties shown in Figure 4b is primarily due to the exposed areas where sulfurization of non-covered Ag NPs occur.

## 4. Conclusions

We reported a simple method for the synthesis of graphene–silver nanoparticle hybrids on HOPG substrates. We showed that in the case of a graphene overlayer, the Ag NPs tend to coalesce and to form larger nanoparticles. STM and STS measurements performed on graphene–Ag NPs revealed charge transfer from silver resulting in the *n*-doping of graphene. We demonstrated by optical reflectance investigations that a graphene overlayer preserves the local surface plasmon resonance properties of Ag NPs for at least three months, although the LSPR is gradually redshifted. We showed by SEM and EDX that graphene can protect Ag NPs from ambient sulfur for more than one year. Nevertheless, with the applied transfer process, only 40–50% of Ag NPs are coated and the observed loss of plasmonic properties is primarily attributed to the spontaneous sulfurization of non-covered NPs. The long-term stability of LSPR could be significantly improved by increasing the total graphene coverage. Such protection of Ag NPs by an atomically thin cover layer can be very useful, for example in LSPR shift-based sensor applications, photocatalysis, or the preparation of advanced substrates for surface-enhanced Raman spectroscopy.
